# Oxidative Transformation of Controlled Substances by Manganese Dioxide

**DOI:** 10.1155/2015/364170

**Published:** 2015-05-19

**Authors:** Webber Wei-Po Lai, Angela Yu-Chen Lin, Sheng-Yao Yang, Ching-Hua Huang

**Affiliations:** ^1^Graduate Institute of Environmental Engineering, National Taiwan University, 71 Chou-Shan Road, Taipei 106, Taiwan; ^2^School of Civil and Environmental Engineering, Georgia Institute of Technology, Atlanta, GA 30332, USA

## Abstract

This study investigated the oxidative transformation of four controlled substances (ketamine, methamphetamine, morphine, and codeine) by synthesized MnO_2_ (*δ*-MnO_2_) in aqueous environments. The results indicated that ketamine and methamphetamine were negligibly oxidized by MnO_2_ and, thus, may be persistent in the aqueous environment. However, morphine and codeine were able to be oxidized by MnO_2_, which indicated that they are likely naturally attenuated in aqueous environments. Overall, lower solution pH values, lower initial compound concentrations, and higher MnO_2_ loading resulted in a faster reaction rate. The oxidation of morphine was inhibited in the presence of metal ions (Mn^2+^, Fe^3+^, Ca^2+^, and Mg^2+^) and fulvic acid. However, the addition of Fe^3+^ and fulvic acid enhanced codeine oxidation. A second-order kinetics model described the oxidation of morphine and codeine by MnO_2_; it suggested that the formation of a surface precursor complex between the target compound and the MnO_2_ surface was the rate-limiting step. Although the target compounds were degraded, the slow TOC removal indicated that several byproducts were formed and persist against further MnO_2_ oxidation.

## 1. Introduction

The presence of pharmaceuticals in aqueous environments is an important environmental issue because conventional wastewater treatment plants (WWTP) do not remove them, resulting in their release into the environment [1, 2]. Controlled substances are one type of pharmaceuticals commonly used in hospitals. Besides their medical applications, controlled substances are also used illicitly and abusively. These substances can be classified as antidepressants, stimulants, and hallucinogens based on their effects on the central nervous system. In addition, these compounds can cause significant toxicity after prolonged exposure because they are chemically and biologically active [3].

Ketamine, methamphetamine, morphine, and codeine are four controlled substances and have been detected in various waterbodies in different countries. For example, they were detected in hospital effluents (maximum concentrations of 10,000, 260, 1,240, and 378 ng/L, resp.), and ketamine, methamphetamine, and codeine were detected in river water in Taiwan (maximum concentrations of 341, 405, and 57 ng/L, resp.) [4, 5]. Boleda et al. [6] reported that morphine and codeine were detected at concentrations of up to 81 and 397 ng/L, respectively, in WWTP effluents in Spain. Hummel et al. [7] investigated the occurrence of morphine and codeine in Germany and found that both of these compounds were present in river water (Rhine water) (78 ng/L; 94 ng/L), WWTP influents (820 ng/L; 540 ng/L), and effluents (110 ng/L; 260 ng/L). In addition, Castiglioni et al. [8] investigated the occurrence of morphine and methamphetamine in Italy and Switzerland, maximum concentrations of 204 and 16 ng/L, respectively, in the WWTP influent and 55 and 4 ng/L, respectively, in the WWTP effluent. The release of these controlled substances into the aqueous environment may endanger aquatic life and result in ecosystem contamination.

Natural attenuation (hydrolysis, redox reaction, sorption, photolysis, and biodegradation) is considered to be one of the most important pathways for removing contaminants from the aqueous environment and is the most economically feasible method for further attenuation of treated wastewater [9]. However, very limited information is currently available regarding the environmental fates of these four controlled substances (ketamine, methamphetamine, codeine, and morphine), and the oxidative transformation of these four controlled substances by manganese oxide (MnO_2_) has not been previously investigated. MnO_2_ is abundant in natural sediments and soils and has been reported to possess a powerful oxidative capacity for removing phenol, aniline, aliphatic amine, and triazine in aquatic environments through oxidation and sorption [10, 11].

The objective of this study was to investigate the oxidative transformation of four controlled substances (ketamine, methamphetamine, morphine, and codeine) by synthesized MnO_2_ (*δ*-MnO_2_) in an aqueous environment. Several critical environmental factors that may affect the MnO_2_ oxidation rate were studied, including the MnO_2_ loading, initial compound concentration, and solution pH. In addition, the effects of cosolutes (metal ions (Mn^2+^, Fe^3+^, Ca^2+^, and Mg^2+^) and natural organic matter) on MnO_2_ oxidation were investigated, and total organic carbon removal and oxidative transformation byproducts were studied.

## 2. Experimental Setup

### 2.1. Chemicals

Morphine, codeine, and ketamine were purchased from Sigma-Aldrich (St. Louis, MO, USA). Methamphetamine was purchased from the US Pharmacopeial Convention (USP). Without any further purification, the purities of all drugs were greater than 95%. All other stock solutions were prepared in deionized water at concentrations of 500–1,000 mg/L and were stored at 4°C in a refrigerator. Other chemicals were purchased from Merck Millipore (Guyancourt, France), Avantor Performance Materials (Phillipsburg, NJ, USA), Sigma-Aldrich (St. Louis, MO, USA), and Nacalai Tesque (Kyoto, Japan) with purities greater than 85% (Table S1 in Supplementary Material available online at http://dx.doi.org/10.1155/2015/364170). The physicochemical properties of ketamine, methamphetamine, codeine, and morphine are listed in Table 1.

### 2.2. Synthesis of Manganese Dioxide

*δ*-MnO_2_ was synthesized according to the methods of Murray [12]. First, 80 mL of 0.1 M KMnO_4_ and 160 mL of 0.1 M NaOH were added to 1.64 L of N_2_-sparged reagent water, and 120 mL of 0.1 M MnSO_4_ was added to the solution while continuously stirring. After the MnO_2_ particles settled, the supernatant solution was replaced with deionized water until the conductivity of the MnO_2_ suspension was less than 2 *μ*S-cm^−1^. The final volume of the MnO_2_ solution was adjusted to 1 L, and then the solution was stored at 4°C.

### 2.3. Chemical Analysis

High-performance liquid chromatography-tandem mass spectrometry (HPLC-MS/MS) was used to analyze the concentrations of the four target controlled substances. The HPLC module consisted of a pump (Agilent 1200 Series Binary Pump), degasser (Agilent 1200 Series Micro Vacuum Degasser), and autosampler (Agilent 1200 Series Autosampler). The analytes were determined using chromatography with a ZORBAX Eclipse XDB-C_18_ column (150 × 4.6 mm, 5 *μ*m particle size) and a flow rate of 1 mL min^−1^. Mobile phases A and B consisted of 0.05% formic acid with 10 mM ammonium acetate in DI water and methanol, respectively.

### 2.4. Oxidation of Controlled Substances

MnO_2_ oxidation experiments were conducted using 250 mL screw-cap amber glass bottles with aluminum foil septa at room temperature (22°C). The solution pH was controlled using a 10 mM acetate buffer (pH 4 and 5), 4-morpholinepropanesulfonic acid (MOPS) (pH 6 and 7), 2-(cyclohexylamino)ethanesulfonic acid (CHES) (pH 9), and a phosphate buffer (pH 9). In addition, NaCl (0.01 M) was added to maintain an appropriate ionic strength.

The volume of the reactor was fixed at 100 mL. The reaction aliquots were collected (1 mL) and quenched using two different methods: the filtration quenching method and the reductant quenching method. In the reductant quenching method, oxalic acid (0.5 g/L) was added to desorb the target compound from the MnO_2_ surface. The filtration quenching method can only detect target compounds dissolved in solution, not those adsorbed on the surface of manganese dioxide.

### 2.5. Analysis of Oxidative Byproducts

To analyze the oxidative byproducts of codeine, an Agilent LC-ESI-MS/MS combined with a ZORBAX Eclipse XDB-C_18_ column (150 × 4.6 mm, 5 *μ*m particle size) was used to perform quantitative analyses. Three steps were required to analyze the byproducts. First, the electron-spray ionization (ESI) detector was operated to obtain a full mass spectrum scan (Q1 scan) ranging from 50 to 600. Then, the selected sample was compared with the control sample to determine the possible oxidative byproduct. Second, the multiple-reaction monitoring transition mode (MRM) was used to optimize the parameters for MS/MS detection, including the declustering potential (DP), collision energy (CE), and collision cell exit potential (CXP). Third, the LC parameters were optimized to separate the analyte, including the mobile phase, flow rate, and injection volume.

## 3. Results and Discussion

Background tests (hydrolysis and adsorption) were performed before the MnO_2_ oxidation experiment. The four controlled substances remained stable in the water at all tested pH conditions (pH 4 and 5 for ketamine and methamphetamine; pH 5, 7, and 9 for morphine; and pH 5, 6, 7, 8, and 9 for codeine) (Figure S1). No obvious differences were observed between the filtration quenching method and the reductant quenching method (Figure S2) and no measurable adsorption by MnO_2_ was observed by the four controlled substances.

**Figure 1:**
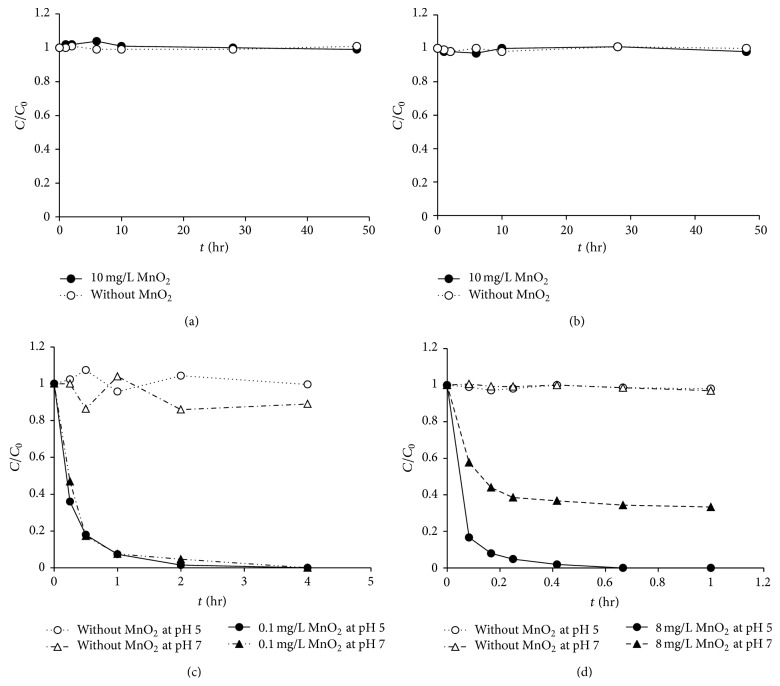
Oxidation of the four controlled substances by MnO_2_: (a) ketamine at pH 5, (b) methamphetamine at pH 5, (c) morphine, and (d) codeine (compound concentration = 100 *μ*g/L, ionic strength = 10 mM).

The MnO_2_ experiments revealed that ketamine and methamphetamine were persistent and were not oxidized by 10 mg/L MnO_2_. However, morphine and codeine were oxidized by MnO_2_, indicating that they may undergo attenuation in natural soil and sediment environments (Figure 1). Based on the kinetic model reported by Zhang et al. [11], the oxidation of morphine and codeine follows second-order kinetics with adsorption as the rate-limiting step. The second-order kinetics of this process are shown in (1), where *k*_1_, *k*_−1_, and *k*_2_ present the adsorption rate constant of the compound by MnO_2_, the desorption rate constant of the compound by MnO_2_, and the electron transfer rate constant, respectively. Because the adsorption reaction is considerably slower than the electron transfer reaction, the amount of target compound (morphine and codeine) adsorbed on MnO_2_ but unreacted would be negligible for detection as observed by the experiments. Consider

(1) $$\begin{array}{c} -\frac{dC}{dt}=\frac{k_{1}k_{2}\left[S_{\text{rxn}}-\left(C_{0}-C\right)\right]C}{k_{-1}+k_{2}}=k^{\prime \prime }\left[S_{\text{rxn}}-\left(C_{0}-C\right)\right]C. \end{array}$$

### 3.1. Effects of pH

**Figure 2:**
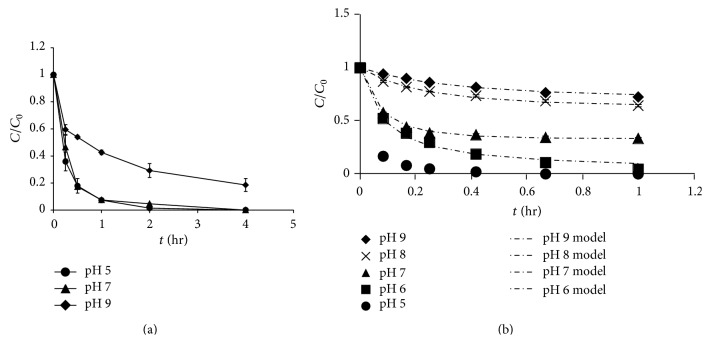
Effect of solution pH on the oxidation of controlled substances by MnO_2_: (a) morphine (with 100 *μ*g/L MnO_2_) and (b) codeine (with 8 mg/L MnO_2_) (morphine and codeine = 100 *μ*g/L, pH = 7, and ionic strength = 10 mM) (the second-order kinetic model developed by Zhang et al. [11] was fitted to describe the codeine oxidation).

An initial environmentally relevant concentration was selected for the four controlled drugs (100 *μ*g/L). Ketamine and methamphetamine remained stable against MnO_2_ oxidation (4 mg/L) at pH 4 and 5 for 48 h (Figure S3). However, the degradation behaviors of morphine and codeine were different from that of ketamine and methamphetamine. In this study, different MnO_2_ loadings were used for morphine and codeine (100 *μ*g/L MnO_2_ for morphine and 8 mg/L MnO_2_ for codeine) because morphine is oxidized by MnO_2_ much more readily than codeine. The results showed that morphine and codeine exhibited lower degradation rates as the solution pH decreased (Figure 2). Morphine was >99%, 91.5%, and 82% degraded at pH 5, 7, and 9, respectively (reaction time = 4 h, MnO_2_ = 100 *μ*g/L). Codeine was >99%, 96%, 67%, 36%, and 28% degraded at pH 5, 6, 7, 8, and 9, respectively (reaction time = 1 h, MnO_2_ = 8 mg/L). Previous studies indicated that the oxidation of compounds by synthesized MnO_2_ is a surface reaction and that the surface charge of MnO_2_ is negative (point of zero charge (PZC) of MnO_2_ was 2.25) [12, 13]. Therefore, the intensity of the negative MnO_2_ charge increased as the pH increased from 5 to 9. This increase caused the MnO_2_ surface to become more hydrophilic and subsequently reduced the number of accessible active sites for the target compound because of obstruction from the nearby water molecules, which further reduced the reaction rate [14]. Lin et al. [15] also demonstrated that the ability of MnO_2_ to oxidize organic compounds is pH-dependent. Furthermore, Stumm and Morgan [16] reported that the MnO_2_ reduction potential decreased from 0.99 V to 0.76 V when the pH increased from 4 to 8. Zhang and Huang [17] also reported that more surface species are produced at lower pH that subsequently react with the reductants. In addition, Klausen et al. [18] indicated that protons are required to dissociate the reaction product Mn(II) from the MnO_2_ (Mn(IV)) mineral surface ((1/2)Mn^IV^O_2(s)_ + 2H^+^ + e^−^⇄(1/2)Mn^2+^ + H_2_O). Therefore, a higher solution pH may result in a lower degradation rate.

### 3.2. Effect of MnO_2_ Loading

**Figure 3:**
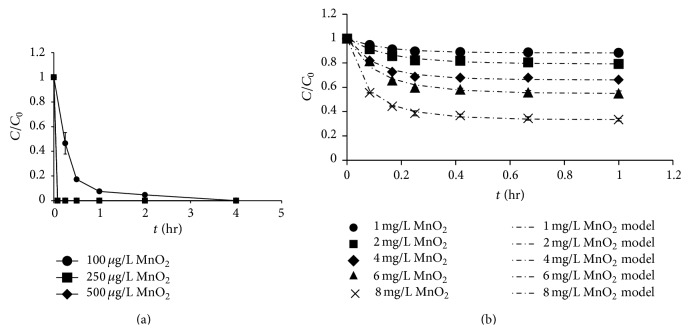
Effect of MnO_2_ loading on the oxidation of controlled substances by MnO_2_: (a) morphine and (b) codeine (morphine and codeine = 100 *μ*g/L, pH = 7, and ionic strength = 10 mM) (the second-order kinetic model developed by Zhang et al. [11] was fitted to describe the codeine oxidation).

The results showed that ketamine and methamphetamine were still present after MnO_2_ oxidation for 48 hrs unless the MnO_2_ loading reached 750 mg/L (Figure S4). High MnO_2_ loading (750 mg/L) indicates that ketamine and methamphetamine are not degraded by natural MnO_2_ in soils and sediments. For morphine and codeine, higher MnO_2_ loadings resulted in a faster oxidation rate (Figure 3). The degradation rates of morphine by 250 and 500 *μ*g/L MnO_2_ were faster than that by 100 *μ*g/L MnO_2_. Similarly, when MnO_2_ increased from 1 mg/L to 8 mg/L, the degradation efficiency of codeine increased from 12% to 65% in 1 h. Zhang et al. [11] reported that a precursor complex between the organic compound and MnO_2_ is formed before electron transfer reaction occurs during the redox reaction. These two steps (precursor complex formation and electron transfer reaction) were hypothesized to be rate-limiting steps and to influence the reactivities of organic compounds and MnO_2_. Thus, the oxidation of organic compounds by MnO_2_ resulted from surface reactions between the MnO_2_ and compounds [10]. Consequently, when the MnO_2_ concentration increased, the potential for contact between the target compounds and MnO_2_ increased and resulted in the reaction. Furthermore, Zhang and Huang [17] reported that higher MnO_2_ concentrations offer more active surface sites, which resulted in an increase in the rate of precursor complex formation.

### 3.3. Effects of the Initial Compound Concentration

**Figure 4:**
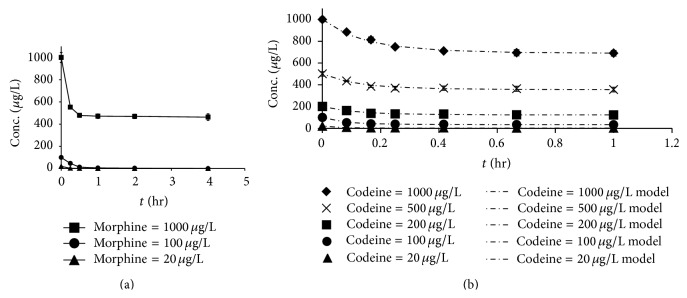
Effect of initial compound concentration on the oxidation of controlled substances by MnO_2_: (a) morphine (with 100 *μ*g/L MnO_2_) and (b) codeine (with 8 mg/L MnO_2_) (pH = 7, ionic strength = 10 mM) (the second-order kinetic model developed by Zhang et al. [11] was fitted to describe the codeine oxidation).

It has been hypothesized that the formation of a surface complex is the rate-limiting step in the oxidation of morphine and codeine by MnO_2_. The formation rate of such a surface complex is affected by the MnO_2_ loading, solution conditions (pH and ionic strength), and the initial concentration of the target compound. In this study, the initial compound concentration (morphine and codeine) range was 20 to 1,000 *μ*g/L. The results showed that increasing compound concentrations resulted in decreased degradation efficiency (Figure 4) when the MnO_2_ loading and pH were fixed. A higher compound concentration could result in self-competition for the fixed number of active sites on the surface of MnO_2_, thereby decreasing the removal rate of morphine and codeine [11].

### 3.4. Effects of Metal Ions

**Figure 5:**
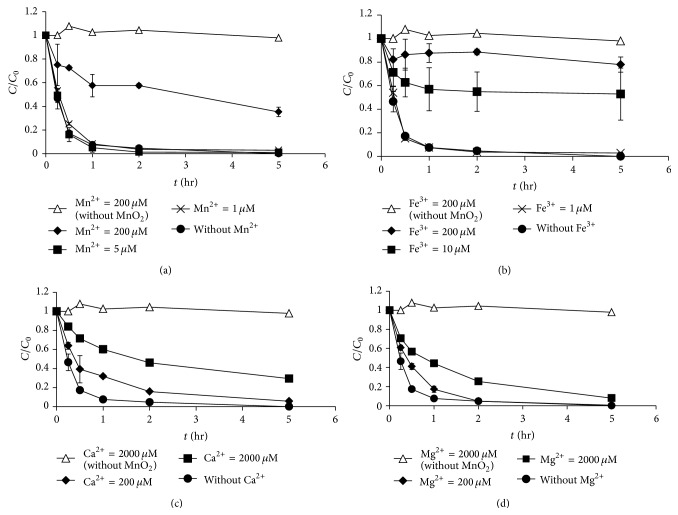
Effect of metal ions on the oxidation of morphine by MnO_2_: (a) Mn^2+^, (b) Fe^3+^, (c) Ca^2+^, and (d) Mg^2+^ (MnO_2_ loading = 100 *μ*g/L, initial morphine concentration = 100 *μ*g/L, pH = 7, and ionic strength = 10 mM).

**Figure 6:**
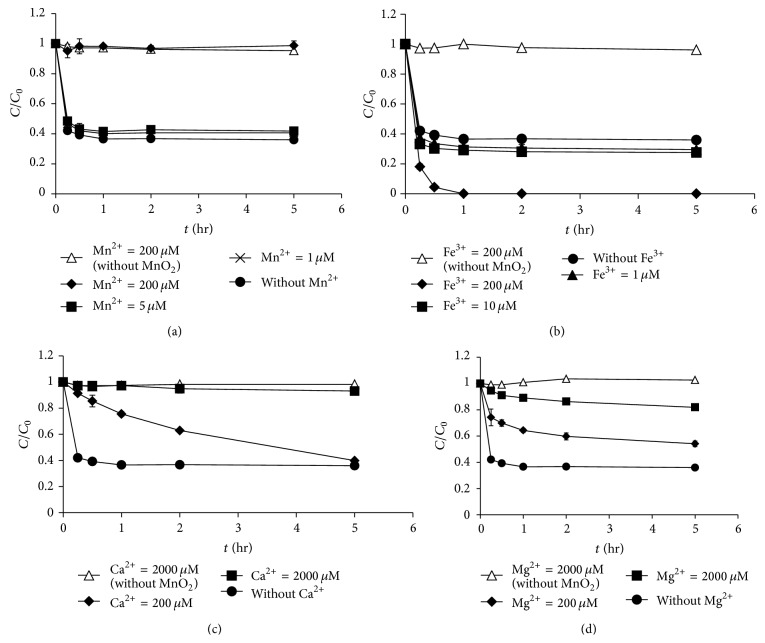
Effect of metal ions on the oxidation of codeine by MnO_2_: (a) Mn^2+^, (b) Fe^3+^, (c) Ca^2+^, and (d) Mg^2+^ (MnO_2_ loading = 8 mg/L, initial codeine concentration = 100 *μ*g/L, pH = 7, and ionic strength = 10 mM).

The effects of metal cations (Mn^2+^, Fe^3+^, Ca^2+^, and Mg^2+^) on the oxidation of morphine and codeine by MnO_2_ were investigated (Figures 5 and 6). Previous studies showed that dissolved cations in real water matrices may inhibit the degradation of compounds by MnO_2_ [11, 17]. Our results showed a similar phenomenon, in which the coexistence of metal ions resulted in an inhibitory effect on the oxidation of morphine and codeine by MnO_2_ (except for the effect of Fe^3+^ on the oxidation of codeine). The degradation of morphine by MnO_2_ was inhibited more when the concentrations of the metal cations (Mn^2+^, Fe^3+^, Ca^2+^, and Mg^2+^) increased. For example, as the Mn^2+^ concentration increased from 1 to 200 *μ*M, the degradation efficiency of morphine decreased from 99.0% to 65.7% in 4 h. However, when the Ca^2+^ concentration increased from 200 to 2,000 *μ*M, the degradation efficiency decreased from 94.3% to 70.6%. Similarly, the degradation efficiency of codeine decreased when the metal cation concentrations (Mn^2+^, Ca^2+^, and Mg^2+^) increased. However, the opposite phenomenon was observed regarding the effects of Fe^3+^ on the degradation of codeine (Fe^3+^ enhanced the degradation efficiency of codeine by MnO_2_). As the Fe^3+^ concentration increased from 1 to 200 *μ*M, the degradation efficiency of codeine increased from 54.6% to 100%. One possible explanation for this result is that the addition of FeCl_3_ at pH 7 could result in coagulation of MnO_2_ particles, which might reduce the codeine concentration in the solution. The induced coagulation is more likely to occur at the higher MnO_2_ loading (8 mg/L) applied for codeine. Further investigations should be performed to understand the detailed mechanisms of Fe^3+^ toward codeine degradation by MnO_2_.

### 3.5. Effects of Natural Organic Matter

**Figure 7:**
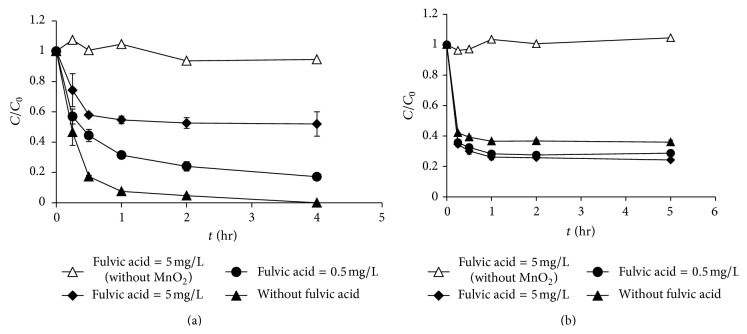
Effect of fulvic acid on the oxidation of (a) morphine (with 100 *μ*g/L MnO_2_) and (b) codeine (with 8 mg/L MnO_2_) (compound concentration = 100 *μ*g/L, pH = 7, and ionic strength = 10 mM).

Natural organic matter (NOM) is present in aqueous environments and is also a factor that may influence the oxidation efficiency of MnO_2_ [18]. In this work, fulvic acid (FA) was used to represent the NOM in the natural environment. The effects of FA on the oxidation of morphine and codeine by MnO_2_ are shown in Figure 7. Results showed that FA decreased the degradation efficiency of morphine, potentially because FA competes with target compounds to the active surface hydroxyl groups of MnO_2_, resulting in slowing the removal of morphine [21]. In contrast, greater concentrations of FA resulted in slightly greater codeine removal. This result implied that FA did not compete with codeine for the same reactivity sites on MnO_2_. Another possibility is that the presence of FA might facilitate some aggregation of the MnO_2_ particles under the experimental conditions used for codeine (with 8 mg/L MnO_2_). In the absence of MnO_2_, FA alone did not react with morphine or codeine (Figure 7), which indicated that the interaction between FA and the target compound (morphine or codeine) did not result in the removal of the target compound.

### 3.6. Total Organic Carbon (TOC) and Oxidative Byproduct Analysis

**Figure 8:**
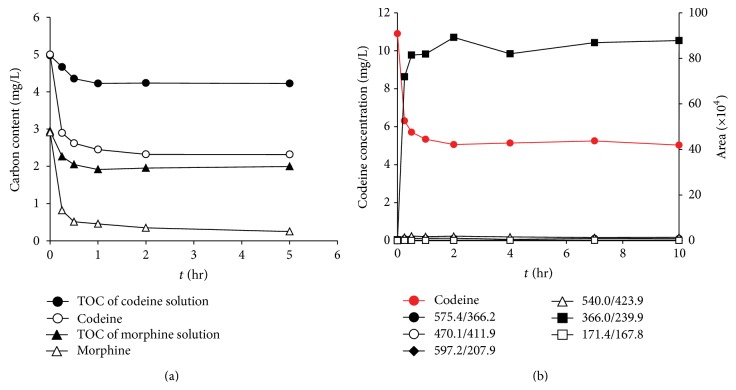
(a) Target compound degradation and TOC removal of codeine and morphine and (b) byproducts of codeine (MnO_2_ = 8 mg/L, pH = 7).

The results obtained for the removal of TOC help determine whether the target compounds were mineralized or transformed into other oxidative transformation byproducts by MnO_2_. According to Figure 8(a), although 93% and 54% of the carbon in morphine and codeine were degraded within 5 h, the TOC removals were only 12% and 33%, respectively. This result indicated that various degradation byproducts of morphine and codeine were formed. The degradation byproducts of codeine were investigated in a preliminary experiment, as shown in Figure 8(b). The signal corresponding to the byproducts did not decrease during the oxidation of codeine by MnO_2_, indicating that these byproducts may be persistent and resistant to oxidation by MnO_2_. In addition, the signal corresponding to the byproducts reached a plateau once the codeine concentration stopped decreasing. This result implied that the formation of byproducts would be affected by the rate of surface complex formation. Among all of the byproducts investigated, the molecular weights of the five byproducts (*m/z* = 575.4, 470.1, 597.2, 540.0, and 366.0) were higher than the parent compound codeine, indicating that these intermediates may be formed by the interaction and combination of the byproducts in the solution. The MS/MS spectra of the oxidative byproducts of codeine are shown in Figure S5. Further work is required to comprehensively investigate the formation of the degradation byproducts and their environmental fate and behavior in aqueous environments.

## 4. Conclusions

This study investigated the oxidative transformation of ketamine, methamphetamine, morphine, and codeine by MnO_2_. Ketamine and methamphetamine were stable against oxidation by MnO_2_ until MnO_2_ loading of 750 mg/L was used, indicating that ketamine and methamphetamine are negligibly degraded by natural MnO_2_ in soils and sediments and may persist in the environment if no other oxidative pathways are involved. Morphine and codeine were oxidized by MnO_2_, which indicated that they can be naturally attenuated in aquatic environments with the presence of MnO_2_. A higher MnO_2_ loading, a lower pH, and a lower compound concentration resulted in a higher efficiency of morphine and codeine oxidation by MnO_2_. The kinetic modeling results showed that the oxidation of morphine and codeine followed second-order kinetics and was limited by the rate of the surface precursor complex formation. The presence of metal ions (Mn^2+^, Fe^3+^, Ca^2+^, and Mg^2+^) and FA inhibited the oxidation of morphine by MnO_2_. Although Mn^2+^, Ca^2+^, and Mg^2+^ suppressed the oxidative efficiency of codeine, Fe^3+^ and FA enhanced its degradation, but more research is needed to elucidate the mechanisms. In addition, although the target compounds were degraded, the slow removal of TOC indicated that several byproducts were formed and were even more persistent against further oxidation.

## Supplementary Material

The Supplementary Material provides additional 1 table and 5 figures: Table S1: Information regarding the chemicals used in this study. Figure S1: Hydrolysis tests of ketamine, methamphetamine, morphine, and codeine under different pH conditions. Figure S2: Comparison of two quenching methods for ketamine, methamphetamine, morphine and codeine. Figure S3: Effect of solution pH on the oxidation of ketamine and methamphetamine. Figure S4: Effect of MnO2 loading on the oxidation of ketamine and methamphetamine. Figure S5: MS/MS spectrum for the byproduct of codeine.

## Figures and Tables

**Table 1:** Physicochemical properties of ketamine, methamphetamine, morphine, and codeine.

	Molecular formula	Molecular weight (g/mol)	pKa	Solubility (mg/mL)	Structure
Ketamine	C_13_H_16_ClNO	237.7	7.5^b^	250	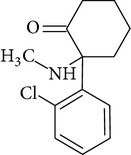

Methamphetamine	C_10_H_15_N	149.2	9.9^a^	NA	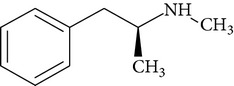

Morphine	C_17_H_19_NO_3_	285.3	9.85^b^ (phenol group)	60	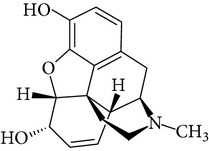

Codeine	C_18_H_21_NO_3_	299.4	9.8^b^	434.8	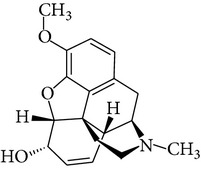

^a^[19], ^b^[20].
