# Supplementary material for: Oxidative Transformation of Controlled Substances by Manganese Dioxide
Source: ScientificWorldJournal. 2015 May 19;2015:364170. doi: 10.1155/2015/364170 (PMC4452481; doi:10.1155/2015/364170)
Supplement: Supplementary file 1 — The Supplementary Material provides additional 1 table and 5 figures: Table S1: Information regarding the chemicals used in this study. Figure S1: Hydrolysis tests of ketamine, methamphetamine, morphine, and codeine under different pH conditions. Figure S2: Comparison of two quenching methods for ketamine, methamphetamine, morphine and codeine. Figure S3: Effect of solution pH on the oxidation of ketamine and methamphetamine. Figure S4: Effect of MnO2 loading on the oxidation of ketamine and methamphetamine. Figure S5: MS/MS spectrum for the byproduct of codeine. [file 364170.f1.docx]

**Table S1** Information regarding the chemicals used in this study.

| Reagent | CAS number | Purity | Source |
| --- | --- | --- | --- |
| ascorbic acid (AA) | 144-62-7 | 99% | Sigma-Aldrich |
| oxalic acid (OA) | 50-81-7 | ≧99% | Sigma-Aldrich |
| 3-(N-morpholino)propanesulfonic acid | 1131-61-2 | ≧99.5% | Sigma-Aldrich |
| *N*-Cyclohexyl-2-aminoethanesulfonic acid | 103-47-9 | ≧99% | Sigma-Aldrich |
| formic acid | 64-18-6 | ≧98% | Sigma-Aldrich |
| morphine sulfate salt pentahydrate | 6211-15-0 | ≧98% | Sigma-Aldrich |
| ketamine hydrochloride | 1867-66-9 | ≧99% | Sigma-Aldrich |
| codeine | 76-57-3 | ≧98% | Sigma-Aldrich |
| sodium persulfate | 7775-27-1 | ≧99% | Sigma-Aldrich |
| sodium phosphate monobasic monohydrate | 7558-80-7 | ≧99% | Sigma-Aldrich |
| acetic acid | 64-19-7 | 100% | Avantor Performance Materials |
| manganese(II) sulfate monohydrate | 10034-96-5 | 99.9% | Avantor Performance Materials |
| methanol | 67-56-1 | ≧99.9% | Avantor Performance Materials |
| sodium chloride | 7647-14-5 | ＞99.5% | Nacalai tesque |
| sodium acetate | 127-09-3 | ≧98% | Nacalai tesque |
| phosphoric acid | 7664-38-2 | 85% | Nacalai tesque |
| potassium hydrogen phthalate | 28726-05 | 98% | Nacalai tesque |
| methamphetamine | 51-57-0 | ≧99.99% | U.S. Pharmacopeial Convention |
| potassium permanganate | 7722-64-7 | ≧99% | Merck Millipore |

(a)

(b)

(c)

(d)

**Figure S1** Hydrolysis tests of (a) ketamine, (b) methamphetamine, (c) morphine, and (d) codeine under different pH conditions (initial compound concentration = 100 μg/L, ionic strength = 10 mM).

(a)

(b)

(c)

(d)

**Figure S2** Comparison of two quenching methods for (a) ketamine (with 10 mg/L MnO_2_ at pH 4), (b) methamphetamine (with 10 mg/L MnO_2_ at pH 4), (c) morphine (with 100 μg/L MnO_2_ at pH 7) and (d) codeine (with 8 mg/L MnO_2_ at pH 7) (initial compound concentration = 100 μg/L, ionic strength = 10 mM).

(a)

(b)

**Figure S3** Effect of solution pH on the oxidation of (a) ketamine and (b) methamphetamine (MnO_2_ loading = 10 mg/L, initial compound concentration = 100 μg/L, ionic strength = 10 mM).

(a)

(b)

**Figure S4** Effect of MnO_2_ loading on the oxidation of (a) ketamine and (b) methamphetamine (initial compound concentration = 100 μg/L, pH = 4, ionic strength = 10 mM).


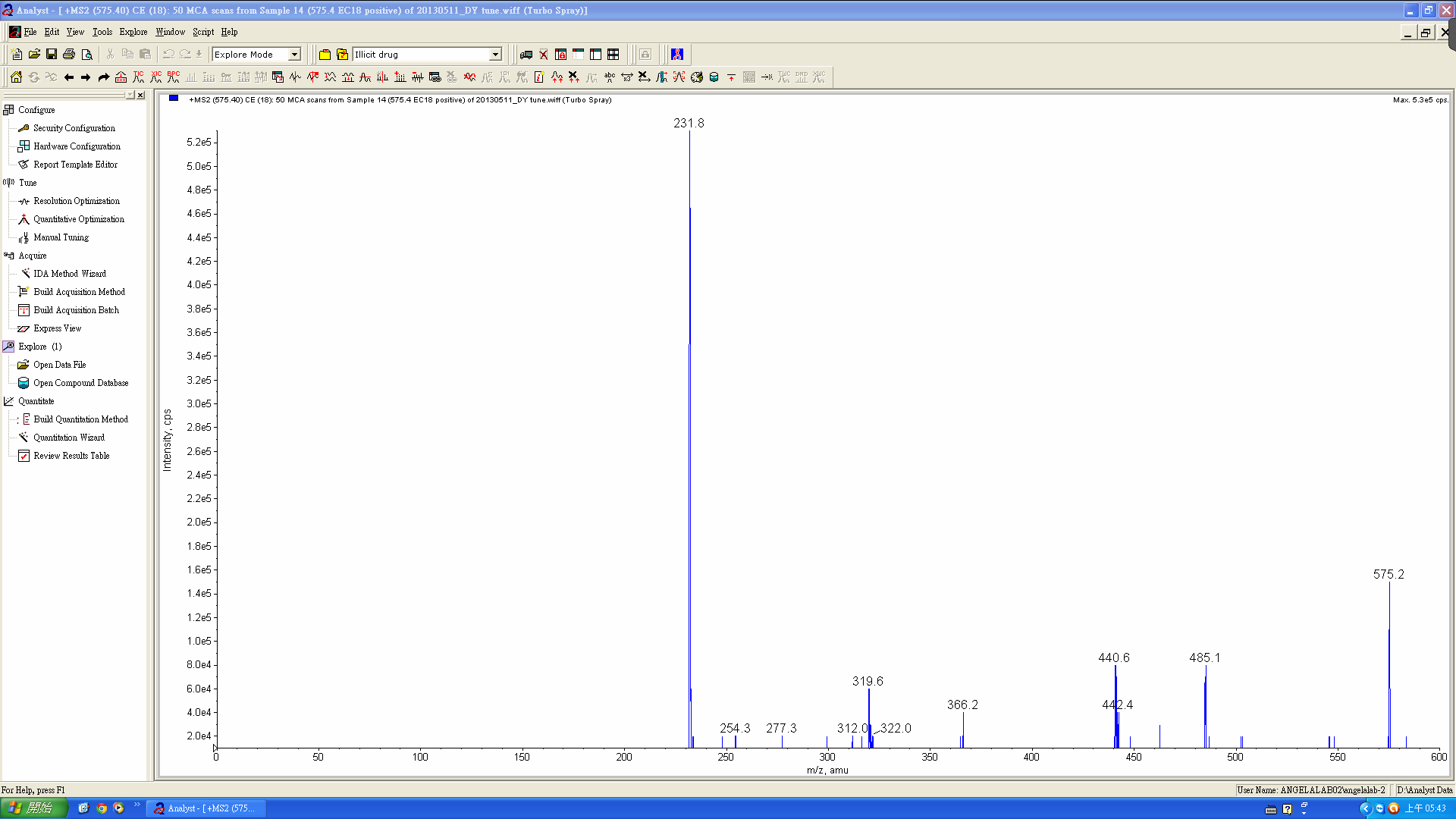


**Figure S5(a)** MS^2^ spectrum for the byproduct of codeine, [M+H] = 575.4.


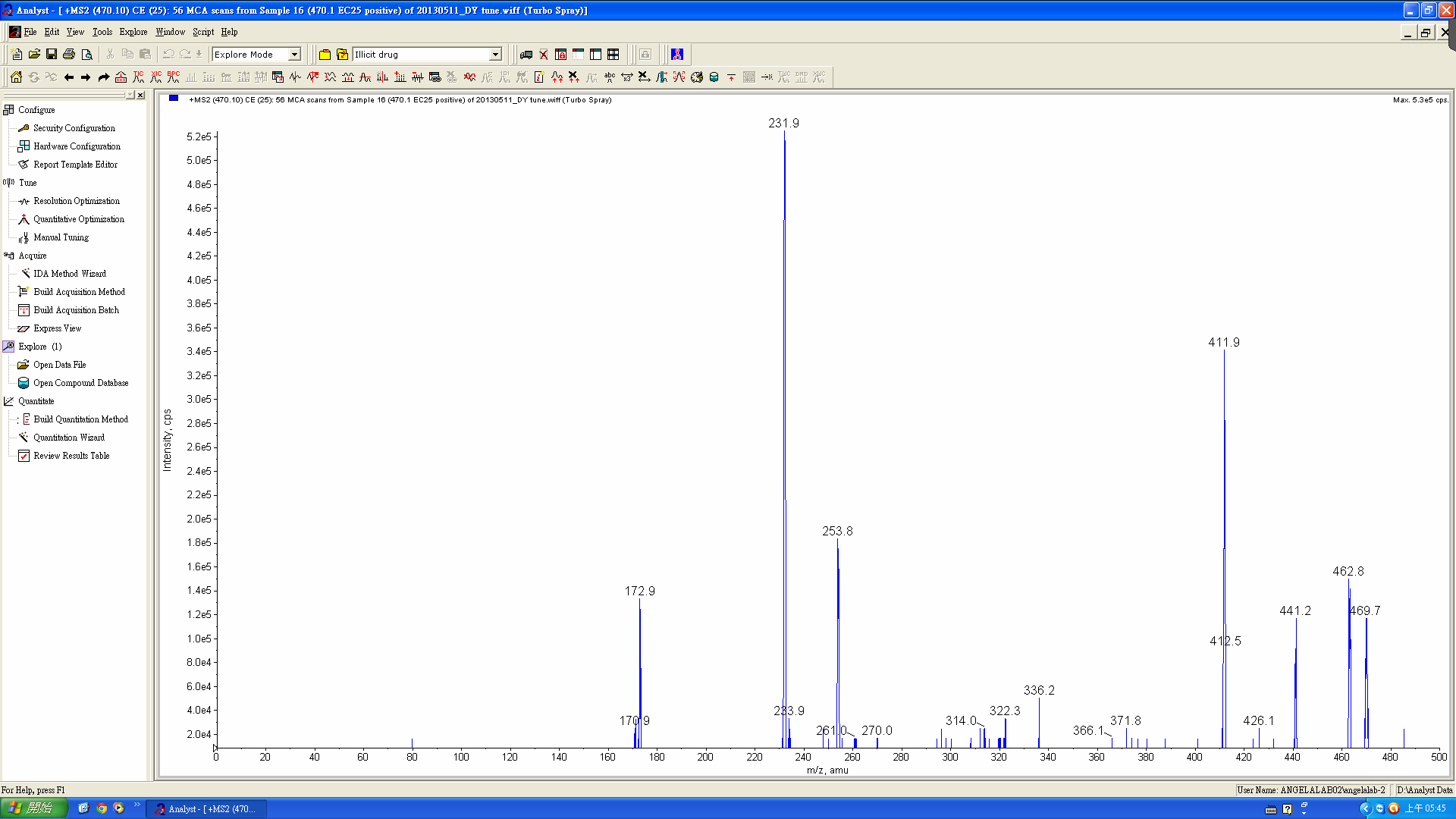


**Figure S5(b)** MS^2^ spectrum for the byproduct of codeine, [M+H] = 470.1.


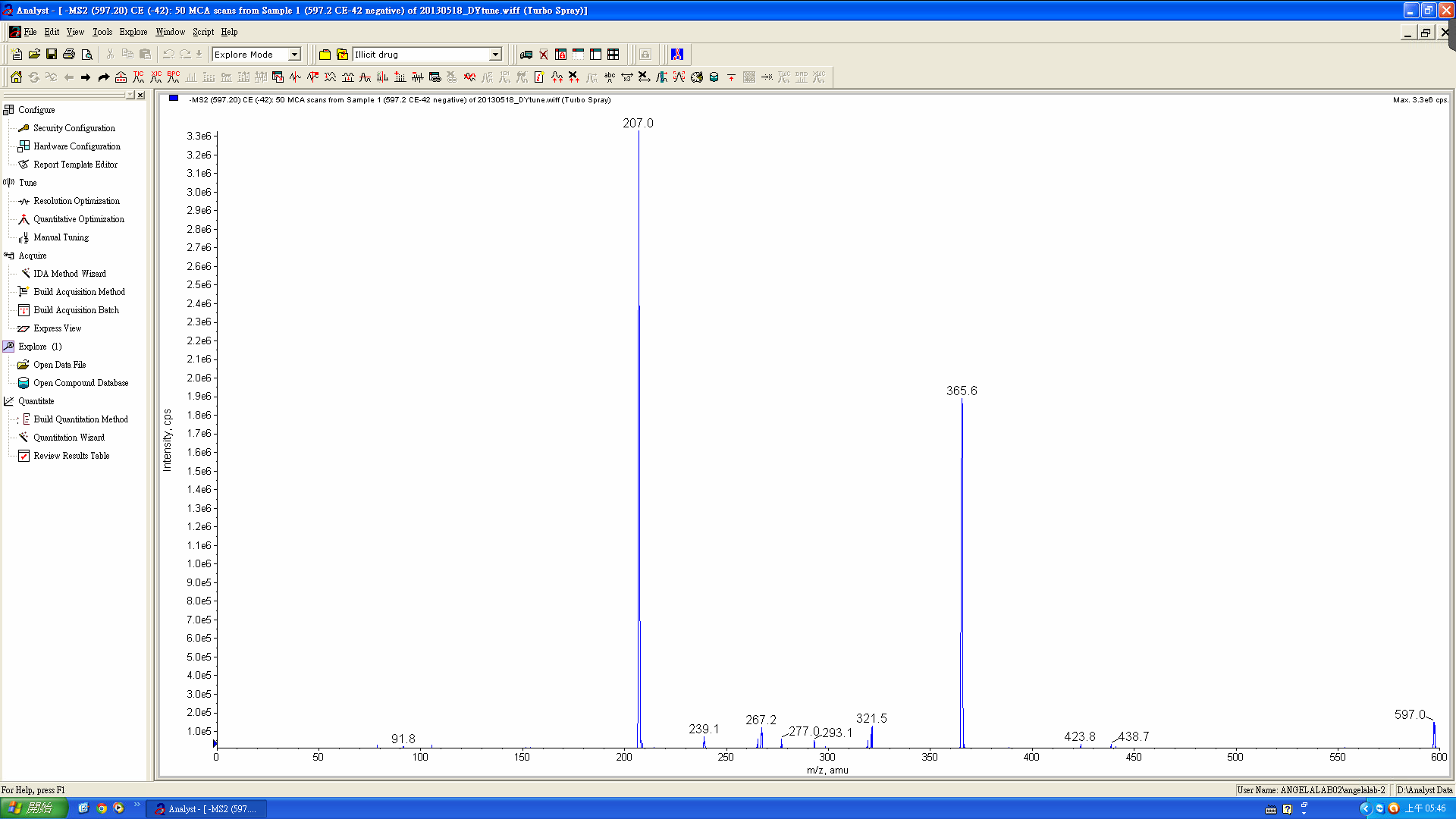


**Figure S5(c)** MS^2^ spectrum for the byproduct of codeine, [M+H] = 597.2.


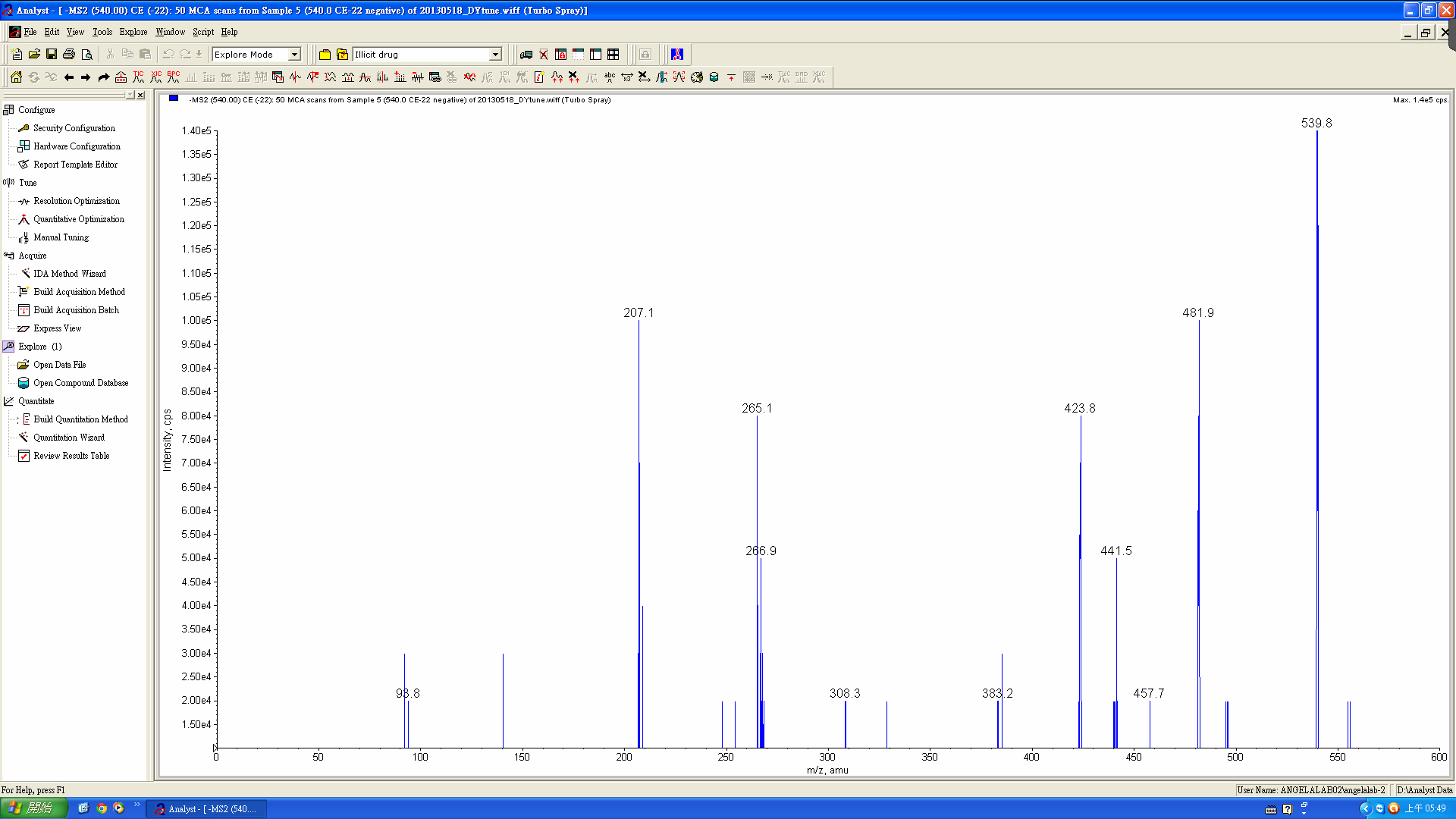


**Figure S5(d)** MS^2^ spectrum for the byproduct of codeine, [M+H] = 540.0.


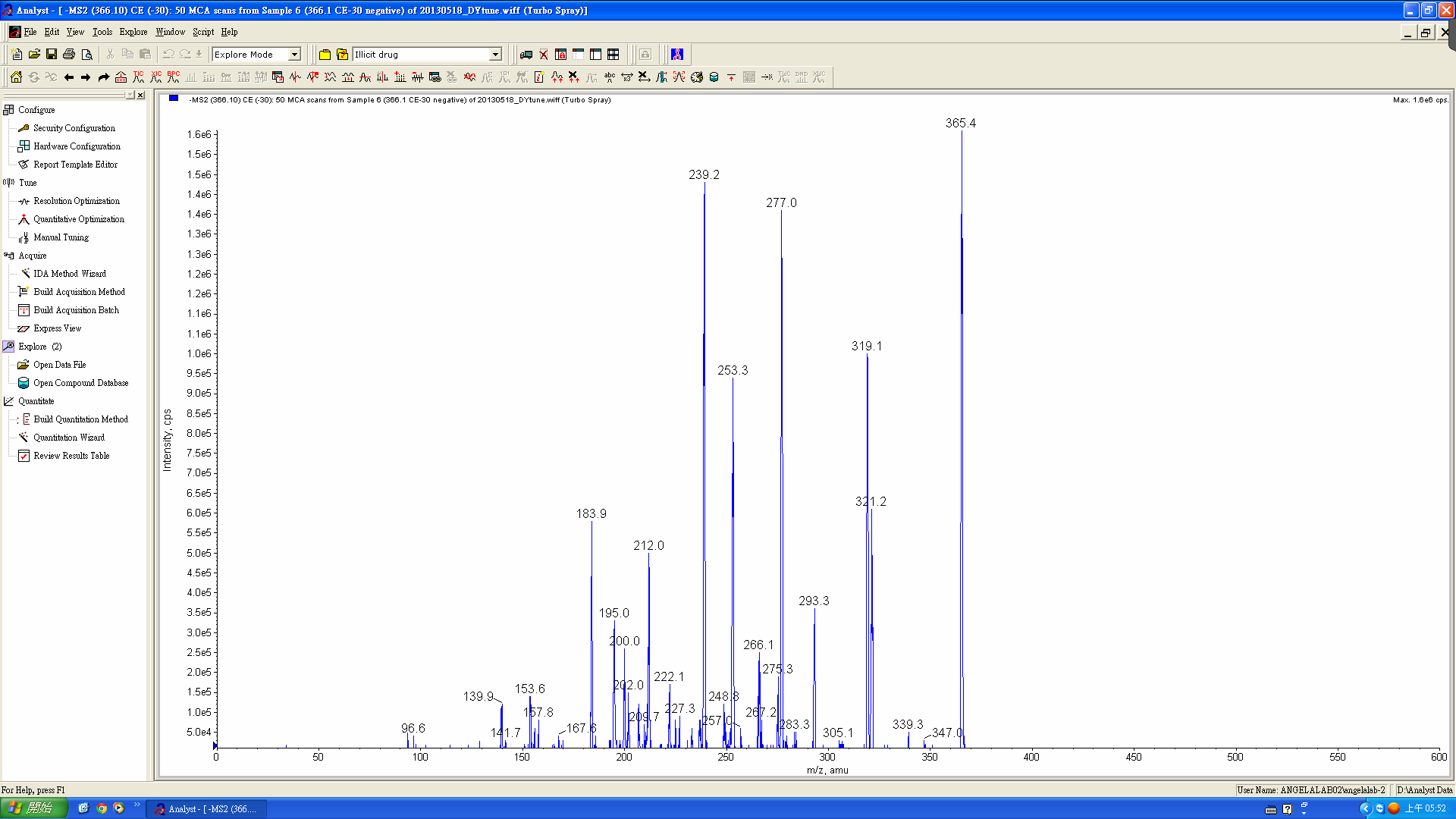


**Figure S5(e)** MS^2^ spectrum for the byproduct of codeine, [M+H] = 366.0.


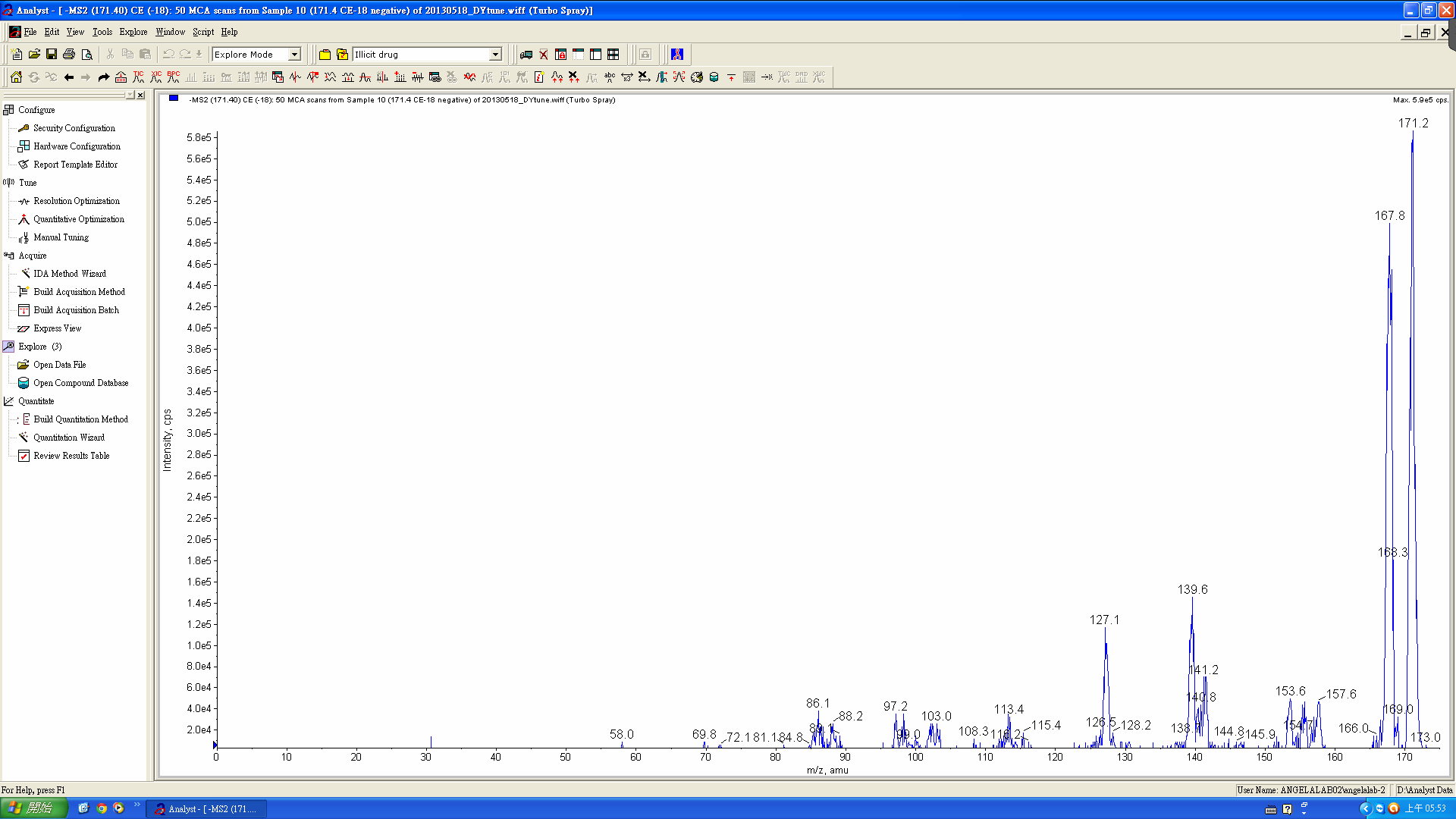


**Figure S5(f)** MS^2^ spectrum for the byproduct of codeine, [M+H] = 171.4.
